# Practical Points

**Published:** 1895-01-19

**Authors:** 


### PRACTICAL POINTS.

*General Hospitals and Infectious Cases.—A correspondent writes to ask what London hospitals, except those of the M.A.B., and the London Fever Hospital, admit fever and infectious cases, adding that in Dublin there are at least nine general hospitals where such cases are received.*

In reply we have to state that *none* of the general hospitals in London admit fever or infectious cases, except cases of typhoid, which experience shows may be treated in general hospitals without any risk, provided proper precautions are taken. The Dublin hospitals have been always nearly a century behind the other hospitals of the world with regard to this question. The continuance of the system is a reflection upon the intelligence and capacity of the authorities responsible for the management of the hospitals, and it is difficult to imagine how it is possible that Dublin should be content to continue this dangerous custom. If there is a medical officer of health, in Dublin, or any health authority worthy of the name, proceedings should be taken against, and penalties imposed upon, the authorities who admit fever and infectious cases into general wards. It is the plain duty of the health authorities to prevent the direct spread of infection, and failure on their part to enforce the law should arouse the community in self-defence and ensure their removal from office and the substitution of others with a higher sense of public duty and greater capacity and character for the fulfilment of the duties which they are appointed to perform.

*A Quarter's Notice.—A hospital matron, who is about to give up her appointment to be married, puts before us the following difficulty. Her committee, having previously accepted a quarter's notice, write through the Secretary to request her to make room for her successor some six weeks before the expiration of the quarter. Is she or is she not entitled to demand full compensation in board and lodging up to the date originally named for her departure from the hospital?*

We have taken legal opinion on this point, and find that if the terms of our correspondent's engagement include the use of rooms and board, and that the committee have accepted her resignation as from a given date, she will be entitled to compensation in the event of their requiring her to leave her rooms and failing to provide board earlier than that date. She will, in fact, be entitled to the emoluments of her office down to the date from which the committee have accepted her resignation.

